# Investigation Into the Relationship Between Sperm Cysteine-Rich Secretory Protein 2 (CRISP2) and Sperm Fertilizing Ability and Fertility of Boars

**DOI:** 10.3389/fvets.2021.653413

**Published:** 2021-04-30

**Authors:** Fenglei Gao, Ping Wang, Kai Wang, Yushan Fan, Yuming Chen, Yun Chen, Chao Ye, Meiying Feng, Li Li, Shouquan Zhang, Hengxi Wei

**Affiliations:** ^1^Guangdong Provincial Key Lab of Agro-animal Genomics and Molecular Breeding, National Engineering Research Center for Breeding Swine Industry, College of Animal Science, South China Agricultural University, Guangzhou, China; ^2^Department of Tropical Agriculture and Forestry, College of Guangdong Agriculture Industry Business Polytechnic, Guangzhou, China; ^3^Technology Department, Guangdong Wen's Foodstuffs Group Co., Ltd., Yunfu, China; ^4^College of Life Sciences, Zhaoqing University, Zhaoqing, China

**Keywords:** CRISP2, sperm, fertilization, fertility, boar

## Abstract

The proteins in the seminal plasma and on the sperm surface play important roles in sperm function and numerous reproductive processes. The cysteine-rich secretory proteins (CRISPs) are enriched biasedly in the male reproductive tract of mammals, and CRISP2 is the sole member of CRISPs produced during spermatogenesis; whereas the role of CRISP2 in fertilization and its association with fertility of boars are still unclear. This study aimed to investigate the relationship between the sperm CRISP2 and boar fertility, and explore its impact sperm fertilizing ability. The levels of CRISP2 protein in sperm were quantified by ELISA; correlation analysis was performed to evaluate the association between CRISP2 protein levels and boar reproductive parameters. Meanwhile, the expression of CRISP2 in boar reproductive organs and sperm, and the effects of CRISP2 on *in vitro* fertilization (IVF) were examined. The results showed that boars with high sperm levels of CRISP2 had high fertility. The protein levels of CRISP2 in sperm were positively correlated with the litter size (*r* = 0.412, *p* = 0.026), the number of live-born piglets (*r* = 0.421, *p* = 0.023) and the qualified piglets per litter (*r* = 0.381, *p* = 0.042). CRISP2 is specifically expressed in the testis and sperm of adult boars, and its location on sperm changed mainly from the post-acrosomal region to the apical segment of acrosome during capacitation. The cleavage rate was significantly decreased by adding the anti-CRISP2 antibody to the IVF medium, which indicates CRISP2 plays a critical role in fertilization. In conclusion, CRISP2 protein is specifically expressed in the adult testis and sperm and is associated with sperm fertilizing ability and boar fertility. Further mechanistic studies are warranted, in order to fully decipher the role of CRISP2 in the boar reproduction.

## Introduction

Boar fertility and sperm fertilizing ability are key factors for improving pig production levels and economic benefits, especially in the modern intensive pig industry where artificial insemination (AI) is widely used (1, 2). Proteins, in both the seminal plasma and on the sperm surface, play important regulatory roles in maintaining sperm motility, fertilizing ability, and sperm-egg interaction and are closely related to the fertility of male animals (3, 4). Recently, proteomics approaches identified candidate protein markers in semen for evaluating male fertility, which can help select superior males and improve the production level in animal husbandry (3, 5).

Cysteine-rich secretory proteins (CRISPs) are members of the CRISP, antigen 5 and pathogenesis-related protein 1 (CAP) superfamily and are enriched biasedly in the male reproductive tract of mammals (6, 7). CRISPs are two domain proteins with an N-terminal CAP domain and a cysteine-rich domain (CRD) at the C-terminus called the CRISP domain. The CRISP domain consists of a hinge region and an ion channel region, and eight disulfide bonds in the hall molecule can stabilize structure of CRISPs (7–9). So far, four CRISPs, CRISP1-4, have been found in mice, and three CRISPs, CRISP1-3, have been found in humans, horses and pigs (6, 9, 10).

CRISP2, known as testis-specific protein 1 (Tpx-1), is the sole CRISP produced during spermatogenesis. CRISP2 is localized in the acrosome and tail of sperm, and is released from the acrosome and reassociated at the equatorial segment during the acrosome reaction (11). The reduced expression of CRISP2 in ejaculated spermatozoa has been reported to decrease pregnancy rates in Holstein bulls (12). CRISP2 is necessary for sperm function and male fertility in mice and humans (7, 11), and CRISP2-knockout mice exhibit subfertility phenotypes with an abnormal sperm function (7, 13). Recently, it has been shown that there is a strong relationship between CRISP2 and human spermatogenesis and infertility (14, 15). The expression of CRISP2 was down-regulated in patients with teratoasthenozoospermia, asthenozoospermia or teratozoospermia (16). Contrary to the knowledge gained from mice and humans, the function of CRISP2 in pig reproduction is poorly characterized except for the mRNA expression in reproductive organs (8, 17).

In this study, the relationship between sperm CRISP2 and boar fertility was investigated, and its function on sperm fertilizing ability and the expression profile in sperm and reproductive tissues were analyzed. The present study may reveal the association between the expression level of CRISP2 sperm and the boar fertility, and provide novel insights about CRISP2 expression and function relative to pig reproduction, which could help to enrich the knowledge of sperm CRISP2 and develop new biomarker of male fertility.

## Materials and Methods

### Ethics Statement

This work was approved by the Ethics Committee on Animal Experimentation of South China Agricultural University. The license number is SYXK (Guangdong) 2019-0136.

### Samples

Thirty-three Yorkshire semen samples and the fertility data for each boar were supplied by Shuitai Pig Farm (Guangdong, China). The protein samples of sperm were extracted immediately after semen collection. The sperm proteins were extracted with a whole protein extraction kit (KeyGEN, Jiangsu, China) according to the manufacturer's instructions. Briefly, the sperm samples were centrifuged at 10,000 rpm for 5 min. Supernatant was discarded and sperm pellet was washed thrice with ice-cold DPBS. The sperm pellet was incubated with ice-cold lysis buffer with 1 mM phenylmethylsulfonyl fluoride (PMSF), and the tubes were incubated on ice for 4 min and vortexed for 30 s for 5 times. After incubation, the tubes were centrifuged at 14,000 rpm at 4°C for 4 min. After centrifugation, supernatant was retained as the sperm protein samples.

The reproductive tissues (at least 3 samples) of immature (3 months old) and adult (24 months old) male and female pigs from Shuitai Farm were collected immediately after slaughter and stored in liquid nitrogen or fixed in 4% paraformaldehyde. Testis, epididymis (distal caput), bulbourethral gland (BUG), prostate, and seminal vesicle gland (SVG) were collected from the males. Ovaries, oviduct, uterine horn, uterine body, and cervix were collected from females. The oocytes and granulosa cells were collected from the ovaries in a local slaughterhouse, and the isolation of granulosa cells was performed according to previous study (18). The total RNA and protein of each sample were extracted by using an RNeasy Mini Kit (Qiagen, Hilden, Germany) and a whole protein extraction kit (KeyGEN), respectively, according to the manufacturer's instructions. Purity and concentration of RNA were measured using a NanoDrop ND-1000 instrument (Thermo Fisher Scientific, Waltham, USA). RNA integrity was evaluated using an Agilent 2100 Bioanalyzer (Agilent, San Jose, USA). For protein extraction from tissue samples, the tissues were homologized in lysis buffer containing 1 mM PMSF and were then subjected to centrifugation (14,000 rpm) for 5 min at 4°C. The supernatant was collected as the protein samples.

The protein levels of samples were measured and diluted to an appropriate concentration with a BCA protein assay kit (KeyGEN) according to the manufacturer's instructions. Briefly, a standard curve (range 0–2,000 μg/mL) was derived with nine points of serial dilution with bovine serum albumin (BSA) and a working reagent. All samples and standard points were replicated three times. The samples (100 μL each) were mixed with 2.0 mL of working reagent and incubated at 37°C for 30 min. After cooling to room temperature, each absorbance difference, which was subtracted by averaged absorbance of blank standard replicates at 562 nm, was measured by a spectrometer, and the absorbance differences were converted to μg/mL via the standard curve. If a protein concentration exceeded the upper limit of the standard curve of 2,000 μg/mL, the sample was diluted until it could be measured within the standard range, and the final concentrate was calibrated considering the dilution factor.

### Enzyme-Linked Immunosorbent Assay (ELISA) Detection and Fertility Correlation Analysis of Sperm CRISP2

The protein levels of CRISP2 of each sample were quantified with a porcine CRISP2 ELISA kit (PG1898, TSZ, USA). The assay range was 18–1,450 pg/mL according to the kit instructions. The relative expression level of CRISP2 protein was obtained by dividing the protein levels of CRISP2 by the total protein content. The correlation between the relative content of CRISP2 and the fertility data was conducted using Pearson correlation analysis.

### *In vitro* Fertilization (IVF)

The IVF experiment was conducted as previously reported (19). Briefly, porcine ovaries were obtained from a slaughterhouse and transported to the laboratory in sterile 0.9% NaCl at 38.5°C within 2 h of slaughter. Oocytes were aspirated from follicles (3–6 mm in diameter) with an 18-gauge needle attached to a disposable syringe. Oocytes covered with multilayers of cumulus cells were selected. Oocytes collected were cultured for 44–6 h and denuded in 1 mg/ml hyaluronidase in DPBS by mechanically pipetting; then, 10–15 oocytes were grouped and transferred to the 50 μl mTBM fertilization medium containing 2.5 mM caffeine and 2 mg/ml bovine serum albumin (BSA; fraction V) covered with mineral oil. The fresh semen provided by the Shuitai Farm was washed three times by centrifugation with DPBS supplemented with 0.1% BSA at 1,500 rpm for 4 min. The spermatozoa pellets were resuspended and diluted to 1 × 10^6^ sperm/ml with mTBM for capacitation in the CO_2_ incubator for 30 min. Then, the capacitated sperm were added to the drop containing oocytes with a final sperm concentration of 1 × 10^5^ sperm/ml and co-incubated for 6 h at 39°C in an atmosphere of 5% CO_2_ in air. After fertilization, the oocytes were washed 3 times and cultured with PZM3 medium at 39°C, 5% O_2_, 5% CO_2_, 90% N_2_, and 100% humidity. The cleavage rate was determined after culturing for 48 h.

The effect of CRISP2 on fertilization was tested by adding the anti-CRISP2 antibody (SAB2501635, Sigma, USA) to the fertilization medium mTBM. Briefly, 2 μl of the antibody was added to 500 μl fertilization medium to a final concentration of 2 μg/ml of anti-CRISP2 antibody. The same volume of dilution medium (20 mM Tris (pH 7.3) + 150 mM NaCl + 0.02% sodium azide + 0.5% BSA) or the IgG were added as controls.

### Reverse Transcriptase PCR and Quantitative Real-Time PCR (qRT-PCR)

The gene expression of *CRISP2* in different reproductive organs of different aged male and female pigs was examined by reverse transcriptase PCR. The primers used in the analysis are presented in Table 1. The PCR conditions were as follows: initial denaturation at 94°C for 5 min, followed by 35 cycles of denaturation at 94°C for 30 s, annealing at 60°C for 30 s and extension at 72°C for 40 s, and a final extension at 72°C for 7 min.

**Table 1:** RT-PCR and qRT-PCR primers.

	**Genes**	**Forward (5^**′**^-3^**′**^)**	**Reverse (5^**′**^-3^**′**^)**	**Product size (bp)**
**RT-PCR**	*GAPDH*	CCACCGTCCAGCGAGAAC	CAGCCGAGGAGGTGAGCC	432
	*CRISP2*	ACTCCCAATGGTGCTGTTTC	ATCCAACGCGGTAAGATGAG	418
**qRT-PCR**	*GAPDH*	GAGATCCCGCCAACATCAAAT	GTTCACGCCCATCACAAACAT	170
	*CRISP2*	TGTACAGAGCAAACAGGGCA	GTTGATTGGCACGGTAGGC	194

The relative expression levels of *CRISP2* in the reproductive organs of male and female pigs at different ages were further verified by qRT-PCR using a SYBR-Green RT-PCR Kit (Thermo Fisher Scientific) in an Applied Biosystems 7900HT Real-time PCR Thermal Cycler (Applied Biosystems, Foster City, USA). *GAPDH* was employed as an internal control, and each sample was analyzed three times. The mean values were calculated using the ΔΔCt method as previously reported (20). The PCR conditions were as follows: initial denaturation at 95°C for 3 min, followed by 40 cycles of denaturation at 95°C for 10 s, annealing at 60°C for 10 s and extension at 72°C for 30 s. The qRT-PCR primers are listed in Table 1.

### Western Blot

The proteins (20 μg) of adult boar reproductive tissues and sperm were separated by SDS-PAGE using 12% (v/v) gels and transferred onto PVDF membranes (Millipore, Billerica, MA, USA). After blocking with 5% non-fat milk for 1 h at room temperature, the membranes were incubated with primary antibodies against CRISP2 (1:1,000; SAB2501635, Sigma, USA) or β-actin (1:1,000; HC201, TransGen Biotech, China) overnight at 4°C. The membranes were washed 3 times for 10 min each with TBST (0.1% Tween 20, 20 mM Tris/HCl, 150 mM NaCl; pH 8.0) and incubated for 1 h with horseradish peroxidase (HRP)-conjugated rabbit anti-goat (1:3,000; E030130-02; Earthox, San Francisco, USA) or goat-anti-mouse (1:3,000; HS201, TransGen Biotech, China) secondary antibodies at room temperature for 1 h. The membranes were incubated for 5 min with the enhanced chemiluminescence (ECL) detection reagent in the dark and then exposed with a Tanon-5200 Imaging System (Tanon, Shanghai, China). β-actin was used as the internal control, and the relative protein expression levels of CRISP2 were analyzed by using ImageJ software (https://imagej.nih.gov/ij/index.html).

### Immunohistochemistry Assay

Immunohistochemical detection of CRISP2 in the adult tissues of testis was carried out on 5 μm tissue sections mounted onto siliconized slides. Briefly, paraffin sections were dewaxed with xylene, rehydrated in a graded series of ethanol, and antigen retrieval was performed by heating at 95°C in 10 mM sodium citrate (pH 6.0). Endogenous peroxidase was quenched with 0.3% H_2_O_2_ in methanol for 15 min at room temperature. After 3 washes in PBS (pH 7.4), the slides were incubated in a blocking solution containing 3% BSA for 30 min at room temperature. Sections were incubated overnight at 4°C with antibodies against CRISP2 (1:150; SAB2501635; Sigma, USA), and the primary antibody replaced with normal IgG diluted was served as a negative control. After washing 3 times in PBS, sections were incubated with HRP-conjugated secondary antibodies for 50 min at room temperature. Then, the sections developed with a DAB chromogenic solution and counterstained with a hematoxylin solution. Sections were dehydrated, cleared, covered with Permount solution (Fisher, NH, USA) and viewed under an Olympus BX53F microscope (Olympus, Japan).

### Immunofluorescence Staining

To evaluate the distribution of CRISP2 in the sperm before and after capacitation, immunofluorescence detection was performed as previously described (21). The sperm before and after *in vitro* capacitation were fixed with 4% paraformaldehyde for 20 min, washed three times with PBS, permeabilized with 0.5% Triton-100 for 10 min, and blocked in 1% BSA (Sigma) for 30 min. The sperm were incubated with a goat anti-CRISP2 antibody (1:200; SAB2501635, Sigma, USA) at 4°C overnight and washed three times in PBS. After that, the sperm were incubated with Alexa Fluor 568-donkey anti-goat IgG (1:100; A-11057, Thermo Fisher) 1 h at 37°C in the dark. The samples were coated onto slides and observed under a fluorescence microscope (BX53F, Olympus).

### Statistical Analysis

All the data analysis was performed using the SPSS 18.0 software (IBM, USA). All the data were expressed as mean ± standard deviation. The correlation between the relative content of CRISP2 and the fertility data was conducted using Pearson correlation analysis. The unpaired Student's *t*-test was performed to assess the significant differences between treatment groups. *P* < 0.05 was considered statistically significant.

## Results

### Correlation Between the Sperm CRISP2 Protein Levels and Boar Reproductive Parameters

The relative content of CRISP2 in sperm of 33 boars was detected by ELISA, and effective data was obtained for 29 boars, because a few ELISA wells showed null data. The sperm CRISP2 protein levels and boar reproductive parameters of 29 boars were shown in Supplementary Table 1. The 29 boars were divided into low CRISP2 and high CRISP2 group based on the median values of the sperm CRISP2 protein levels. The correlation analysis listed in Table 2 showed that the protein levels of sperm CRISP2 were significant positive correlation with the boar breeding parameters of litter size (*r* = 0.412, *p* = 0.026), live-born piglets per litter (*r* = 0.421, *p* = 0.023) or qualified piglets per litter (*r* = 0.381, *p* = 0.042), but not with parturition rate (*r* = 0.029, *p* = 0.880) or boar fecundity (*r* = 0.315, *p* = 0.096).

**Table 2:** Correlation analysis between the content of CRISP2 in sperm and the boar reproductive parameters.

**Protein**	**Reproductive parameters**	**Pearson correlation**	***P-*value**
		**coefficient (*r*)**	
Sperm CRISP2	Litter size	0.412	0.026
	No. live-born piglets/litter	0.421	0.023
	No. qualified piglets/litter	0.381	0.042
	Parturition rate	0.029	0.880
	Boar fecundity^#^	0.315	0.096

# *Fecundity equals litter size multiplied by the parturition rate. The number of boars is n = 29, bred 1,842 sows in total*.

To further analyze the correlation between sperm CRISP2 protein levels and the reproductive capacity of boars and to even explore their feasibility as biomarkers for screening boars with high fertility, we ranked the boars corresponding to their protein levels of sperm CRISP2 and divided them into 2 groups: the high CRISP2 group (*n* = 14) and the low CRISP2 group (*n* = 15). The reproductive parameters of the boars are shown in Table 3. There is no significant difference in the number of sows bred (low CRISP2 group: 977 vs. high CRISP2 group: 865) and parturition rate (low CRISP2 group: 92.95 ± 1.51% vs. high CRISP2 group: 93.26 ± 1.22%) between low CRISP2 group and high CRISP2 group. The litter size (low CRISP2 group: 12.18 ± 0.26 vs. high CRISP2 group: 13.08 ± 0.17), live-born piglets per litter (low CRISP2 group: 11.57 ± 0.27 vs. high CRISP2 group: 12.61 ± 0.20), qualified piglets per litter (low CRISP2 group: 10.39 ± 0.26 vs. high CRISP2 group: 11.18 ± 0.17) and boar fecundity (low CRISP2 group: 11.32 ± 0.33 vs. high CRISP2 group: 12.21 ± 0.28) in the CRISP2 group were significantly higher than that in the low CRISP2 group. These results indicated that CRISP2 might play critical roles in the sperm fertilizing ability or boar fertility, and might have the potential to serve as a biomarker for selecting high fertility boars.

**Table 3:** Effect of sperm CRISP2 on boar reproductive performance.

**Items**	**Low CRISP2**	**High CRISP2**
No. of boars	14	15
No. of sows bred	977	865
CRISP2 relative content (10^−7^)	3.26 ± 0.34	14.85 ± 1.48^**^
Litter size	12.18 ± 0.26	13.08 ± 0.17^**^
Live-born piglets/litter	11.57 ± 0.27	12.61 ± 0.20^**^
Qualified piglets/litter	10.39 ± 0.26	11.18 ± 0.17^*^
Parturition rate (%)	92.95 ± 1.51	93.26 ± 1.22
Boar fecundity	11.32 ± 0.33	12.21 ± 0.28^*^

* *p < 0.05;*

** *p < 0.01*.

### Effect of CRISP2 on *in vitro* Fertilization

The effect of the CRISP2 protein on the cleavage rate of *in vitro* fertilization was indirectly investigated by adding the anti-CRISP2 antibody to the fertilization medium during *in vitro* fertilization. As shown in Table 4, the cleavage rate in anti-CRISP2 group (50.37 ± 1.94%) was significantly lower than that of the control group (59.53 ± 2.54%) and the IgG group (57.81 ± 2.19%), which suggests that CRISP2 plays a critical role in the process of fertilization.

**Table 4:** Effect of anti-CRISP2 antibodies on the cleavage rate of *in vitro* fertilization.

**Groups**	**No. of oocytes**	**No of cleaved**	**Cleavage rate/%**
Control	324	192	59.53 ± 2.54^a^
IgG	248	143	57.81 ± 2.19^a^
Anti-CRISP2	261	131	50.37 ± 1.94^b^

*The experiment included 6 replicates. Different letters in the same column indicate significant differences, P < 0.05*.

### CRISP2 Expression in the Reproductive Organs of Pigs

**Figure 1:**
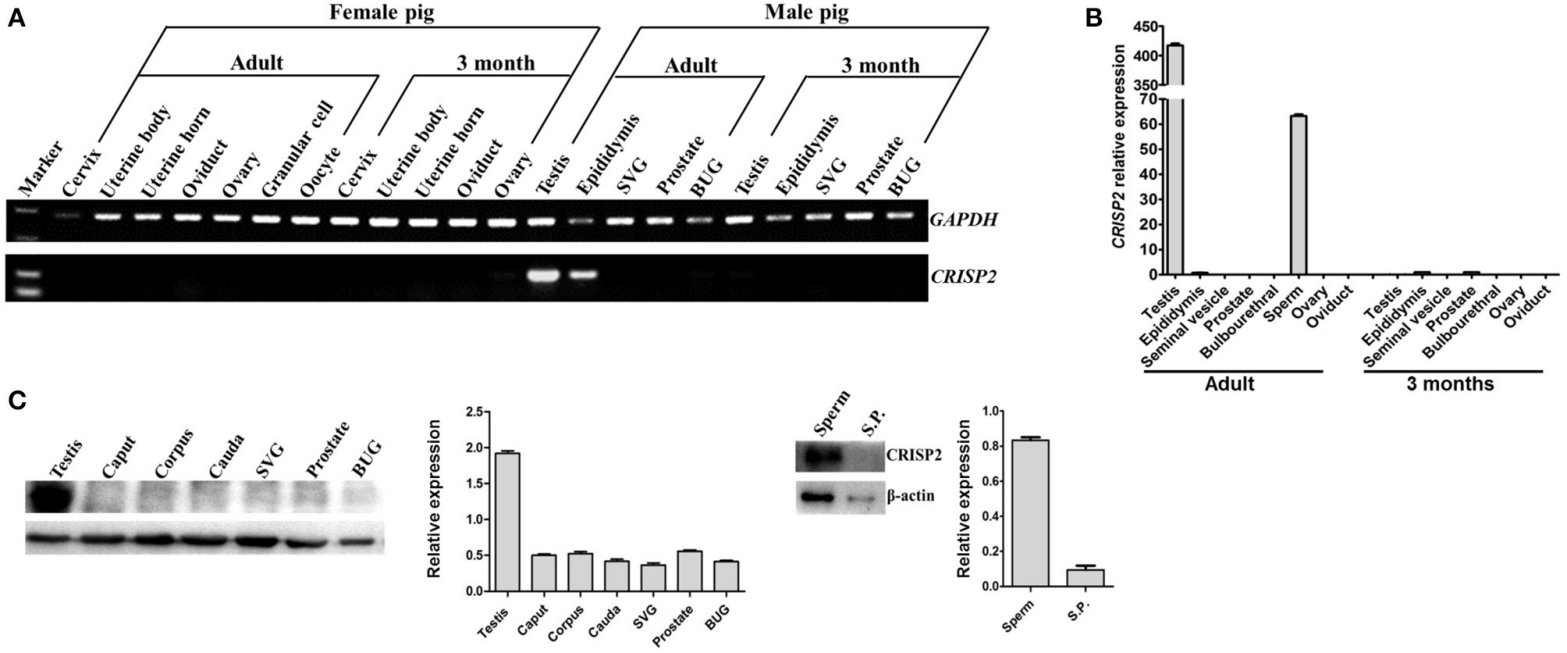
The expression of CRISP2 in the reproductive tissues or cells of male and female pigs with different ages. **(A)** Reverse transcriptase PCR detection of *CRISP2* gene expression in the male and female reproduction systems. **(B)** qRT-PCR detection of *CRISP2* gene expression in male reproductive tissues of the adult and 3 months old pigs. **(C)** Western blot detection of CRISP2 expression in male reproductive tissues of the adult pigs. SVG, seminal vesicle gland; BUG, bulbourethral gland; S.P., seminal plasma.

To elucidate the potential role of CRISP2 in the sperm fertilizing ability and boar fertility, the mRNA expression of *CRISP2* in the reproductive organs of male and female pigs with different ages was detected by reverse transcriptase PCR. As shown in Figure 1, *CRISP2* was expressed specifically in the testis and epididymis of adult boars, and no expression was detected in the reproductive tissues examined from the female pigs (Figure 1A).

To verify the *CRISP2* expression patterns detected by reverse transcriptase PCR, qRT-PCR was conducted on the expression levels of *CRISP2* in the testis, epididymis, SVG, prostate, bulbourethral gland, sperm, ovary and oviduct of adult, and 3-month-old pigs. The results showed that *CRISP2* mRNA was highly expressed in adult testis and sperm (Figure 1B).

The protein level expression of CRISP2 in the boar reproductive organs and semen were further detected by Western blot. The results showed that CRISP2 was mainly expressed in the testis and sperm (Figure 1C).

### Immunohistochemical Analysis of CRISP2 in the Testis of Adult Boars

**Figure 2:**
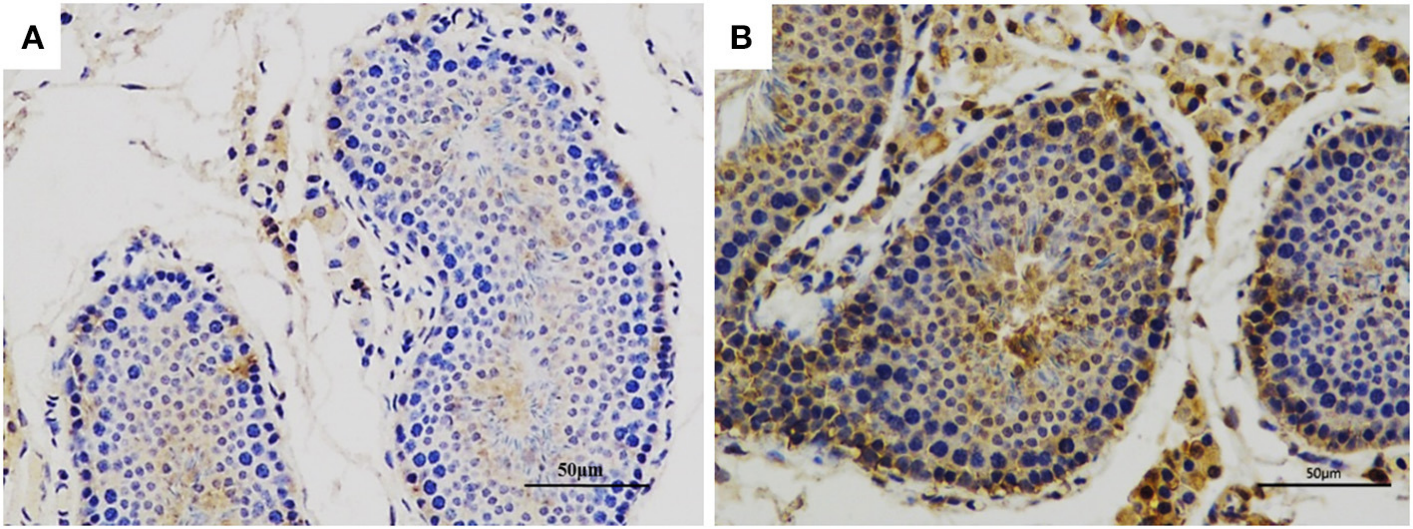
Immunohistochemical analysis of CRISP2 in the testis of adult boars. **(A)** The negative control (IgG control). **(B)** The CRISP2 immunohistochemistry of adult testis. The brown region represents the distribution of target proteins.

The distribution of CRISP2 in the testis tissues was detected by immunohistochemical analysis. The results showed that the CRISP2 protein was expressed in the cytoplasm of spermatogonia, spermatocytes, and sperm cells in the seminiferous tubules (Figure 2).

### Immunofluorescence Detection of CRISP2 in Sperm Before and After Capacitation

**Figure 3:**
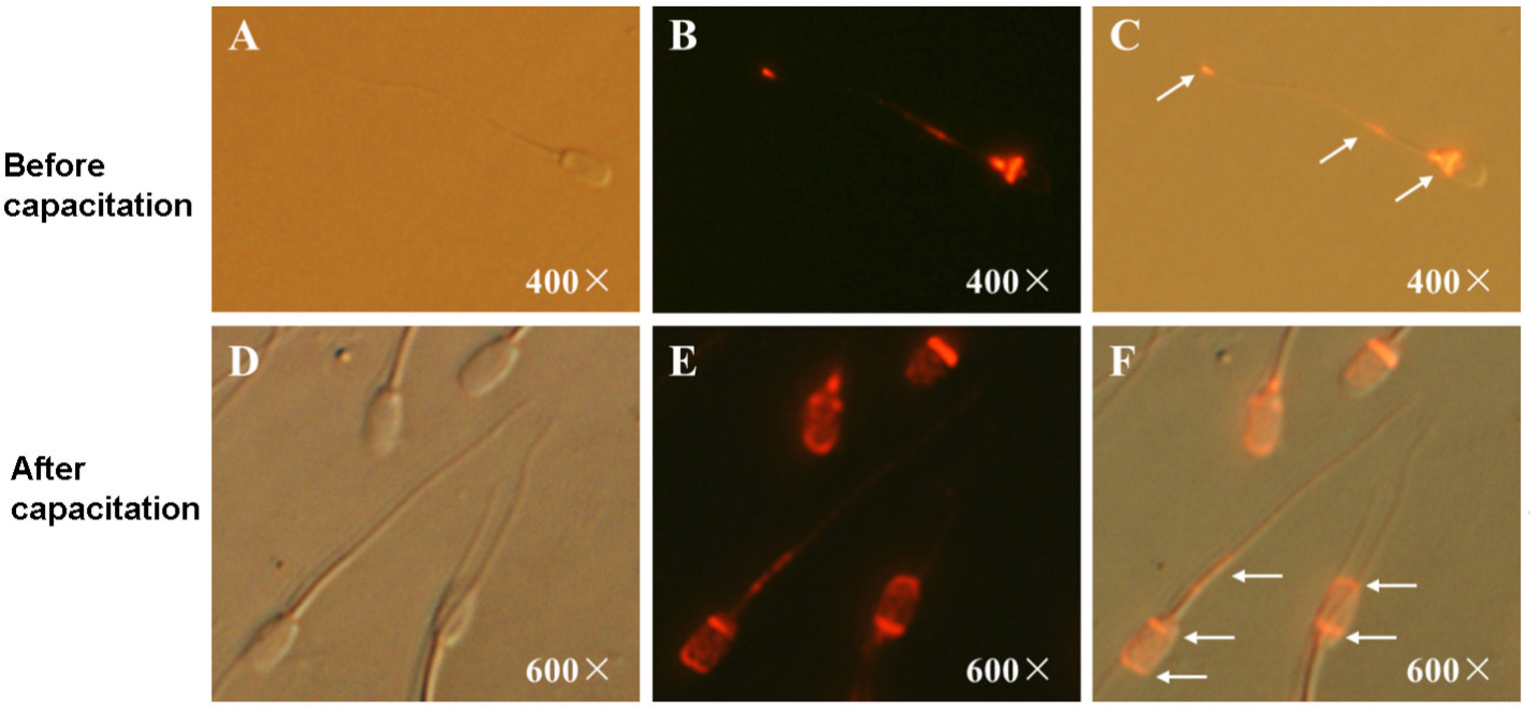
Immunofluorescent staining of CRISP2 in sperm before and after capacitation. **(A–C)** Representative images of the immunofluorescent staining of CRISP2 in sperm before capacitation; **(A)** images taken under light microscope, **(B)** images taken under fluorescent scope; **(C)** merged images. **(D–F)** Representative images of the immunofluorescent staining of CRISP2 in sperm after capacitation; **(D)** images taken under light microscope, **(E)** images taken under fluorescent scope; **(F)** merged images. The white arrow indicates the distribution of the target proteins.

To gain further insight into the function of CRISP2 in fertilization, immunofluorescence detection of CRISP2 before and after sperm capacitation was carried out. As shown in Figure 3, CRISP2 is mainly distributed in the post-acrosomal region, neck, and tail of sperm before capacitation and relocated to the apical segment and posterior of the acrosome and to the middle piece of the tail after sperm capacitation. The number of CRISP2-staining sperm in the apical segment and posterior of the acrosome and middle piece of the tail after capacitation was significantly higher than that before capacitation (before capacitation: 20.63 ± 2.22% vs. after capacitation: 70.23 ± 2.15%, *P* < 0.001).

## Discussion

The present study for the first time showed that the protein level of sperm CRISP2 were positively correlated with the reproductive parameters of litter size, the number of live-born piglets and qualified piglets per litter. Furthermore, the expression profile and the localization of CRISP2 in the reproductive tissues and sperm provide evidence that CRISP2 may play critical roles in sperm fertilizing ability and boar fertility.

CRISPs are members of the CAP superfamily and are expressed specifically in the male reproductive tract in mammals (6, 9). CRISP2 is the sole CRISP produced in the testis during spermatogenesis, and it is specifically localized in the acrosome and tail of sperm. Consistently, our results showed that CRISP2 was expressed specifically in the testis and epididymis of adult boars. CRISP2 is essential for sperm function and male fertility in mice and humans (7, 11). Decreased CRISP2 protein level in sperm was associated with human male infertility (14–16). Studies from Lim et al., indicated that CRISP2 was a quantitative determinant of the ability of sperm to undergo the acrosome reaction, and optimal CRISP2 production was necessary for maximal fecundity in mice (7). Consistently, our results revealed that boars with high levels of sperm CRISP2 protein in boars were associated high reproductive performance.

Although the possible roles of sperm CRISP2 in sperm-oocyte interactions have been investigated in mice and humans (7, 11, 22, 23), the role of CRISP2 and its molecular function in male fertility are still poorly understood, especially in boars. CRISP2-deficient mouse lines show that appropriate CRISP2 expression is necessary for optimal sperm and male fertility (7). Recent studies have shown that sperm CRISP2 is mainly distributed at the acrosome and the tail of wild-type sperm (7, 24, 25). Sperm CRISP2 is associated with the anterior and posterior of the acrosome in capacitated sperm, and CRISP can be released from the acrosome and relocated to the equatorial segment during the acrosome reaction, thereby participating in sperm-egg interaction (11). Our results showed that CRISP2 is mainly distributed in the post-acrosomal region, neck and tail of sperm before capacitation and relocated to the apical segment and posterior of the acrosome and to the middle piece of the tail after sperm capacitation, which suggest that this process may be associated with sperm-oocyte interaction in the pigs. It has been reported that CRISP2 is involved in the calcium flow through ryanodine receptors and the effect may be associated with CatSper, the main calcium channel in sperm, which is vital for sperm motility and male fertility (26, 27).

Busso et al. (23) reported that the anti-CRISP2 antibody significantly decreased the percentage of penetrated eggs of *in vitro* fertilization through a specific participation at the sperm-egg fusion (23). Consistently, our experiments showed that the anti-CRISP2 antibody, significantly decreased the cleavage rate of *in vitro* fertilization in pigs (Table 4). Our study further found that CRISP2 is mainly distributed in the posterior of the acrosome, neck and tail of boar intact sperm and relocated to the anterior and posterior of the acrosome and the tail middle piece of capacitated sperm (Figure 3), which is similar but not entirely consistent with the reports in humans and mice mentioned above. The mRNA of *CRISP2* has been reported to be expressed specifically in boar testis (8). Our results by qRT-PCR and western blot showed that CRISP2 was highly expressed in the sperm and testis of adult boars but not in the ovaries and oviducts of female pigs.

There are several limitations in the present study. Firstly, the present study used the polyclonal CRISP2 antibody to perform the CRISP2 immunohistochemistry of adult testis, which may lead to the non-specific bindings in the testis outside the seminiferous tubes. Future studies may use more specific monoclonal CRISP2 antibodies to confirm its distribution in the testis. Secondly, the sample size in the present study was small, and further studies with large sample size should be considered. Thirdly, the present study only determined effects of CRISP2 antibody on the cleavage rate, and future studies may consider evaluate the effects of CRISP2 antibody on different stages of fertilization.

## Conclusion

In summary, our experiments revealed that the testis and sperm-specific CRISP2 is associated with sperm fertilizing ability and boar fertility and that sperm CRISP2 has the potential to serve as a fertility biomarker. Further mechanistic studies are warranted, in order to fully decipher the role of CRISP2 in the boar reproduction.

## Data Availability Statement

The original contributions presented in the study are included in the article/Supplementary Material, further inquiries can be directed to the corresponding author/s.

## Ethics Statement

This work was approved by the Ethics Committee on Animal Experimentation of South China Agricultural University. The license number is SYXK (Guangdong) 2019-0136.

## Author Contributions

HW conceived the study and drafted the manuscript. FG, PW, KW, YF, and YunC performed the experiments. YumC, CY, and MF analyzed the data. LL and SZ prepared the figures and tables. All authors approved the manuscript for submission.

## Conflict of Interest

CY was employed by the Guangdong Wen's Foodstuffs Group Co., Ltd., company. The remaining authors declare that the research was conducted in the absence of any commercial or financial relationships that could be construed as a potential conflict of interest.

## Abbreviations

AI: artificial insemination
BUG: bulbourethral gland
CAP: CRISP, antigen 5 and pathogenesis-related protein 1
CRD: cysteine-rich domain
CRISP2: cysteine-rich secretory protein 2
SVG: seminal vesicle gland
Tpx-1: testis-specific protein 1.
