# Supplementary material for: Investigation Into the Relationship Between Sperm Cysteine-Rich Secretory Protein 2 (CRISP2) and Sperm Fertilizing Ability and Fertility of Boars
Source: Front Vet Sci. 2021 Apr 30;8:653413. doi: 10.3389/fvets.2021.653413 (PMC8119884; doi:10.3389/fvets.2021.653413)
Supplement: Supplementary file 1 [file Table_1.docx]

**Supplemental Table S1. Sperm CRISP2 protein content and boar fertility**

| Animal Number | Total protein concentration（μg/mL） | Sperm CRISP2 protein concentration (μg/mL) | CRISP2 relative content | Litter size | Number of live-born piglets/litter | Number of qualified piglets/litter | Number of breeding sows | Number of farrowing sows | Parturition rate (%) | Boar fecundity |
| --- | --- | --- | --- | --- | --- | --- | --- | --- | --- | --- |
| Y15-021505 | 54.02 | 6.26×10^-5^ | 1.16×10^-6^ | 13 | 11 | 10 | 182 | 178 | 97.8 | 11.02 |
| Y15-032005 | 235.96 | 7.21×10^-5^ | 3.06×10^-7^ | 12 | 11 | 10 | 229 | 226 | 98.69 | 10.71 |
| Y15-062705 | 89.36 | 1.69×10^-4^ | 1.89×10^-6^ | 13 | 12 | 10 | 99 | 97 | 97.98 | 11.58 |
| Y16-008303 | 256.24 | 5.10×10^-4^ | 1.99×10^-6^ | 13 | 12 | 11 | 127 | 124 | 97.64 | 11.68 |
| Y16-010203 | 161.64 | 2.30×10^-5^ | 1.42×10^-7^ | 14 | 13 | 11 | 71 | 67 | 94.37 | 11.96 |
| Y16-012803 | 289.47 | 1.07×10^-4^ | 3.68×10^-7^ | 13 | 12 | 11 | 57 | 53 | 92.98 | 10.89 |
| Y16-017801 | 263.63 | 1.46×10^-4^ | 5.54×10^-7^ | 12 | 11 | 9 | 105 | 103 | 98.1 | 10.56 |
| Y16-017803 | 270.13 | 8.70×10^-5^ | 3.22×10^-7^ | 12 | 10 | 9 | 120 | 117 | 97.5 | 9.8 |
| Y16-037103 | 176.82 | 1.29×10^-4^ | 0.73×10^-7^ | 13 | 11 | 11 | 46 | 42 | 91.3 | 10.37 |
| Y16-037105 | 319.95 | 2.32×10^-5^ | 7.24×10^-8^ | 13 | 11 | 10 | 22 | 18 | 81.82 | 9.27 |
| Y16-049401 | 206.68 | 7.50×10^-5^ | 3.63×10^-7^ | 14 | 12 | 11 | 72 | 67 | 93.06 | 11.14 |
| Y16-049505 | 198.21 | 2.32×10^-4^ | 1.17×10^-6^ | 15 | 13 | 11 | 38 | 36 | 94.74 | 11.84 |
| Y16-054607 | 139.95 | 2.62×10^-4^ | 1.87×10^-6^ | 14 | 13 | 11 | 25 | 25 | 100 | 12.64 |
| Y16-054901 | 149.56 | 2.17×10^-4^ | 1.45×10^-6^ | 14 | 12 | 11 | 61 | 59 | 96.72 | 12.08 |
| Y16-055805 | 159.64 | 1.36×10^-4^ | 8.53×10^-7^ | 15 | 13 | 11 | 22 | 20 | 90.91 | 11.77 |
| Y16-061301 | 113.69 | 4.07×10^-5^ | 3.58×10^-7^ | 15 | 13 | 12 | 60 | 58 | 96.67 | 12.65 |
| Y16-061403 | 120.96 | 5.44×10^-5^ | 4.50×10^-7^ | 14 | 13 | 11 | 29 | 28 | 96.55 | 12.17 |
| Y16-062101 | 91.53 | 3.54×10^-5^ | 3.87×10^-7^ | 12 | 11 | 10 | 32 | 30 | 93.75 | 9.84 |
| Y16-063201 | 135.8 | 2.32×10^-5^ | 1.71×10^-6^ | 14 | 13 | 12 | 35 | 32 | 91.43 | 11.94 |
| Y16-066803 | 278.75 | 8.89×10^-5^ | 3.19×10^-7^ | 14 | 12 | 11 | 61 | 58 | 95.08 | 11.84 |
| Y16-075801 | 80.92 | 1.48×10^-4^ | 1.83×10^-6^ | 14 | 14 | 12 | 15 | 14 | 93.33 | 12.67 |
| Y16-076801 | 145.52 | 1.46×10^-4^ | 1.00×10^-6^ | 16 | 15 | 13 | 33 | 28 | 84.85 | 12.61 |
| Y16-077601 | 212.7 | 1.43×10^-4^ | 6.70×10^-7^ | 15 | 14 | 13 | 36 | 34 | 94.44 | 13.06 |
| Y16-077901 | 86.26 | 2.34×10^-4^ | 2.72×10^-7^ | 15 | 13 | 12 | 41 | 35 | 85.37 | 11.41 |
| Y16-083401 | 186.31 | 2.75×10^-5^ | 1.48×10^-7^ | 15 | 13 | 11 | 31 | 26 | 83.87 | 10.81 |
| Y16-198803 | 197.92 | 7.86×10^-4^ | 3.97×10^-7^ | 14 | 12 | 11 | 34 | 30 | 88.24 | 10.29 |
| Y16-792805 | 254.31 | 9.79×10^-5^ | 3.85×10^-7^ | 11 | 10 | 9 | 100 | 99 | 99 | 10.1 |
| Y16-915101 | 248.21 | 3.19×10^-4^ | 1.29×10^-6^ | 14 | 13 | 10 | 29 | 25 | 86.21 | 10.86 |
| Y16-915509 | 256.88 | 5.00×10^-4^ | 1.95×10^-6^ | 15 | 13 | 12 | 48 | 41 | 85.42 | 11.38 |
